# Intercolony variations in movement patterns and foraging behaviors among herring gulls (*Larus argentatus*) breeding in the eastern Wadden Sea

**DOI:** 10.1002/ece3.4167

**Published:** 2018-07-06

**Authors:** Leonie Enners, Philipp Schwemmer, Anna‐Marie Corman, Christian C. Voigt, Stefan Garthe

**Affiliations:** ^1^ Research and Technology Centre (FTZ) University of Kiel Büsum Germany; ^2^ Leibniz Institute for Zoo and Wildlife Research (IZW) Berlin Germany

**Keywords:** foraging strategy, GPS tracking, *Larus argentatus*, pellet, stable isotope analysis

## Abstract

Herring gulls (*Larus argentatus*) are opportunistic predators that prefer to forage in the intertidal zone, but an increasing degree of terrestrial foraging has recently been observed. We therefore aimed to analyze the factors influencing foraging behavior and diet composition in the German Wadden Sea. Gulls from three breeding colonies on islands at different distances from the mainland were equipped with GPS data loggers during the incubation seasons in 2012–2015. Logger data were analyzed for 37 individuals, including 1,115 foraging trips. Herring gulls breeding on the island furthest from the mainland had shorter trips (mean total distance = 12.3 km; mean maximum distance = 4.2 km) and preferred to feed on the tidal flats close to the colony, mainly feeding on common cockles (*Cerastoderma edule*) and shore crabs (*Carcinus maenas*). In contrast, herring gulls breeding close to the mainland carried out trips with a mean total distance of 26.7 km (mean maximum distance = 9.2 km). These gulls fed on the neobiotic razor clams (*Ensis leei*) in the intertidal zone, and a larger proportion of time was spent in distant terrestrial habitats on the mainland, feeding on earthworms. *δ* ^13^C and *δ* ^15^N values were higher at the colony furthest from the mainland and confirmed a geographical gradient in foraging strategy. Analyses of logger data, pellets, and stable isotopes revealed that herring gulls preferred to forage in intertidal habitats close to the breeding colony, but shifted to terrestrial habitats on the mainland as the tide rose and during the daytime. Reduced prey availability in the vicinity of the breeding colony might force herring gulls to switch to feed on razor clams in the intertidal zone or to use distant terrestrial habitats. Herring gulls may thus act as an indicator for the state of the intertidal system close to their breeding colony.

## 1 INTRODUCTION

**Figure 1:**
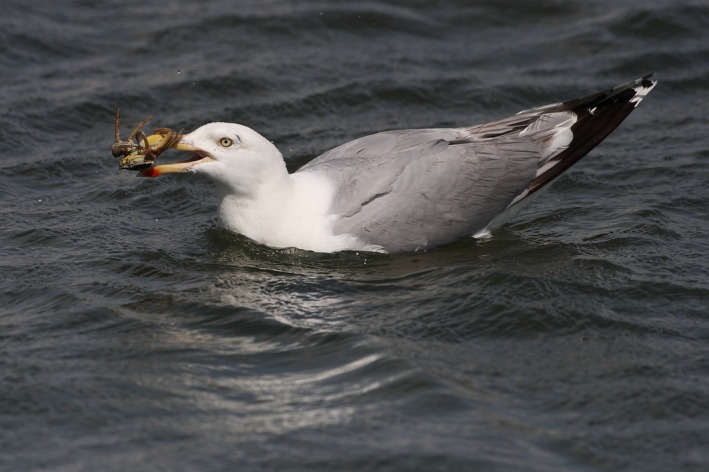
Adult herring gull with prey. Photograph credit: Sven Sturm

Spatial movements and the foraging ecology of seabirds provide important information on food availability and potential habitat changes. Herring gulls (*Larus argentatus*), Pontoppidan 1763, are flexible and opportunistic top predators (Figure 1). In the Wadden Sea World Heritage Site, they mainly forage on intertidal flats, feeding on bivalves and crustaceans (Camphuysen, 2013; Camphuysen & Gronert, 2012; Dernedde, 1993; Kubetzki & Garthe, 2003). Breeding herring gulls at the coast thus showed tide‐dependent foraging patterns (Mendel et al., 2008; Sibly & McCleery, 1983).

Individuals from some colonies also spend a large amount of time foraging in terrestrial habitats on earthworms or anthropogenic refuse (Kim & Monaghan, 2006; Sibly & McCleery, 1983; van Donk, Camphuysen, Shamoun‐Baranes, & van der Meer, 2017). Similar observations have been made for lesser black‐backed gulls (*Larus fuscus*), previously known as a predominantly marine species, but which is increasingly adopting a dual foraging strategy utilizing both marine and terrestrial habitats (Isaksson, Evans, Shamoun‐Baranes, & Akesson, 2016), possibly as a result of food depletion at sea (Garthe et al., 2016; Votier et al., 2004). Furthermore, a multi‐colony study of lesser black‐backed gulls based on individual movement patterns revealed that foraging behaviors also differed between neighboring colonies (Corman, Mendel, Voigt, & Garthe, 2016).

Former studies of foraging behavior and food composition in herring gulls were mainly based on visual observations and pellet analysis (e.g., Kim & Monaghan, 2006; Kubetzki & Garthe, 2003; Pierotti & Annett, 1991; Steenweg, Ronconi, & Leonard, 2011), but GPS data loggers and stable isotope analysis now allow the collection of information on individual movement patterns and foraging behaviors (e.g., Shamoun‐Baranes, Bouten, Camphuysen, & Baaij, 2011; Votier et al., 2010; Wilson et al., 2002). Within‐colony variation in individual movement patterns was recently demonstrated for roof‐nesting herring gulls, based on GPS data (Rock et al., 2016). A multi‐colony approach, based on pellets and stable isotope data of chicks, showed that the breeding success of herring gulls in Scotland depended on the foraging habitat. A higher proportion of intertidal prey sources led to a higher breeding success (O’Hanlon, McGill, & Nager, 2017).

Herring gulls are central‐place foragers (Isaksson et al., 2016; Steenweg et al., 2011) that depend on profitable foraging habitats near their breeding grounds. Prey depletion may imply long flight distances with higher trip costs and an increased risk of nest predation (Morris & Black, 1980; Pierotti & Annett, 1991). Prey availability in the vicinity of the breeding place is thus a “key” factor determining foraging success (Boersma & Rebstock, 2009; Isaksson et al., 2016; Rogers, Piersma, & Hassell, 2006). Tidal flats are visited by herring gulls, as well as by other gull species and a variety of waders, but are only temporarily available for foraging because of tidal water coverage, and previous studies demonstrated both inter‐ and intraspecific competition on tidal flats (Tiedemann & Nehls, 1997).

The German Wadden Sea is characterized by a fine spatial mosaic of different intertidal habitats (e.g., mud flats, sand flats, tidal creeks), with resulting changes in benthos composition and sediment characteristics within small distances. We carried out a multi‐colony approach to compare individual movement patterns and foraging behaviors of herring gulls from neighboring breeding colonies within this highly dynamic system.

The aim of this study was to determine the relationships among individual habitat and prey use by herring gulls and the colony's distance from the mainland, tidal stage, time of day, and sex. We combined data of the diet of gulls at the colony level (stable isotope and pellet analyses) with information on the individual level (GPS loggers) to provide a comprehensive assessment of intercolony differences in individual foraging behaviors during incubation.

## 2 MATERIALS AND METHODS

### 2.1 Study area

**Figure 2:**
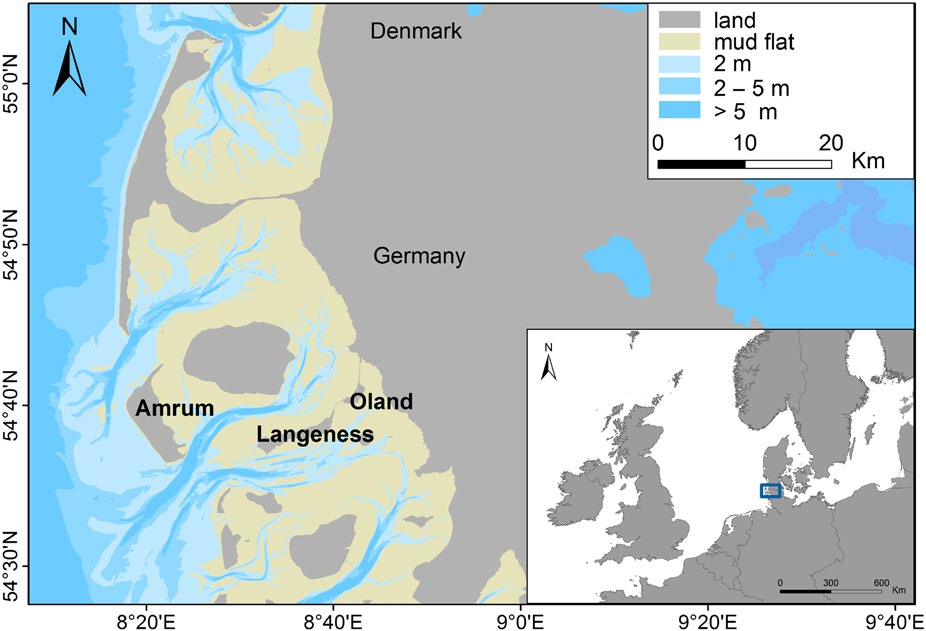
Study area—Oland, Langeness, Amrum. Inserted map: location of the study area within northern Europe

The study was conducted on the islands of Oland (54°40′39″N, 8°42′14″E; 2 km² total area), Langeness (54°38′26″N, 8°37′2″O; 11.5 km²), and Amrum (54°39′6″N, 8°20′11″E; 20.5 km²) in the German North Sea, from 2012 to 2015 (Figure 2). All these islands are located in the Wadden Sea World Heritage Site and are surrounded by tidal flats. The islands host breeding colonies of herring gulls (breeding pairs: Oland, *n* = 300; Langeness, *n* = 640; Amrum, *n* = 700) and represent a geographical gradient from close to the mainland to the outermost intertidal flats adjacent to the open sea. Oland is located closest to the mainland (2.5 km), followed by Langeness (9 km) and Amrum (23 km) (Figure 2). Oland and Langeness are connected to the mainland by an artificial dam.

### 2.2 Experimental setup

Sixty‐three herring gulls were caught on their nests during incubation using walk‐in traps (Oland, *n* = 27; Langeness, *n* = 9; Amrum, *n* = 27). Each bird was ringed, weighed, and equipped with a GPS data logger. Two different types of devices were used. (a) The CatLog‐S (Catnip Technologies, Hong Kong SAR, China; *n* = 47) was attached to the bases of the four innermost tail feathers using textile adhesive TESA tape (Wilson et al., 1997), and birds equipped with these devices were recaptured at the end of the incubation period to remove the devices and download the data. The loggers recorded the date, time, geographical position, and velocity every 2 min. (b) E‐obs Bird Solar 28 g loggers (e‐obs GmbH, München, Germany; *n* = 16) were solar powered and were attached to the back of the gulls using a body harness. Two straps were passed in front of and behind the wings and connected in the middle of the sternum, similar to Thaxter et al. (2014). The data could be downloaded via an ultra‐high frequency connection, and the birds therefore did not need to be recaptured. Date, time, geographical position, velocity, and accelerometer data were recorded at different intervals, depending on the battery status; the devices recorded data every 2–3 min when optimally charged, and at a maximum of every 30 min at lower battery levels. The total masses of the CatLog‐S and e‐obs devices, including the tape or harness, were 28.1 and 39.8 g, respectively, accounting for 3.0% and 3.9%, respectively, of the average body mass of all equipped herring gulls (mean body mass ± standard deviation [*SD*], CatLog‐S: 941 g [±135 g]; e‐obs: 1,017 g [±105 g]), which was within the commonly tolerable limits of 3%–5% of additional body mass (Barron, Brawn, & Weatherhead, 2008; Kenward, 2001) that can be attached to birds. Possible device effects were assessed by monitoring the behavior of the tagged gulls throughout the study period using telescopes.

Blood samples (maximum 0.3 ml) from the ulnar vein were taken from 22 individuals during recapture of the equipped birds and from an additional 20 herring gulls for stable isotope analysis. Furthermore, pellets were collected in the studied colonies.

To analyze the prey availability benthos and sediment samples were taken at foraging hotspots identified from the logger data and at control areas (hotspots: *n* = 322 replicates; control: *n* = 297 replicates).

### 2.3 Dietary analysis

Prey composition was compared among the three colonies by analyzing the collected pellets and blood stable isotope levels. The predominant foraging habitat used by the gulls (*δ* ^13^C, marine vs. terrestrial) and the trophic level of the prey (*δ* ^15^N) were determined by analysis of *δ* ^13^C and *δ* ^15^N values in the blood (Barrett et al., 2011; Hobson & Clark, 1993; Inger & Bearhop, 2008). The blood samples were centrifuged to separate serum and red blood cells, and the diet during the active logger period was determined based on red blood cell isotope levels. The samples were freeze‐dried, and 0.3–0.5 mg of each sample was burned under chemically pure helium gas in an elemental analyzer (FlashEA, ThermoFisher Scientific Bremen, Germany). The resulting gases were then routed via a ConFlo III (ThermoFisher Scientific, Bremen, Germany) to a stable isotope ratio mass spectrometer (Delta V Advantage mass spectrometer; ThermoFisher Scientific) to measure the ratios of light to heavy stable nitrogen and carbon isotopes. Stable isotope values were expressed in delta (*δ)* notation as the deviation from international standards in ‰ (air nitrogen for N and V‐PDB for carbon), according to the following equation: *δX* = [(*R*_sample_/*R*_standard_) − 1] × 1,000; where *X* is ^13^C or ^15^N and *R* is the corresponding ratio ^13^C/^12^C or ^15^N/^14^N. The analytical precision was >0.3‰ (1 *SD*) for stable carbon and 0.2‰ for nitrogen isotopes. Stable isotope analysis of blood samples was conducted at the Leibniz Institute for Zoo and Wildlife Research, Berlin, Germany.

A total of 461 fresh pellets were collected during the incubation periods, and prey items were identified visually to the lowest possible taxon following the method described by Duffy and Jackson (1986). The pellets represented the diet over the preceding 2–3 days. Pellet analysis is known to be biased due to the different digestibilities of different prey items (Barrett et al., 2011; González‐Solís, Oro, Pedrocchi, Jover, & Ruiz, 1997; Kubetzki & Garthe, 2003). We therefore also examined the pellets for shell fragments, earthworm and polychaete bristles, polychaete jaws, fish otoliths and vertebrae, and mammal bones and jaws. To avoid a bias by comparing presence and absence of prey items only, the frequencies of each dietary component were analyzed semi‐quantitatively, as described in Garthe and Scherp (2003) and Schwemmer, Schwemmer, Ehrich, and Garthe (2013). Based on a value of 1 for each pellet, we estimated the main prey items for each pellet considering their digestibility, energy content, and biomass (data on energy content were used from literature, e.g., Cummins & Wuycheck, 1971; own unpublished data). Furthermore, prey items were also classified into three categories: sea, mudflat, and land, according to their origin.

We took benthos and sediment core samples at foraging hotspots identified from the logger data and at control areas, for subsequent habitat analysis. Important foraging sites were selected according to the frequency of visits and duration of time (>30 min) spent within the site. Control areas were sampled randomly at a distance of 300–500 m from the identified hotspots. A total of 200 stations with usually three replicates each (hotspots: *n* = 322 replicates; control: *n* = 297 replicates) were sampled using a corer (diameter: 11.7 cm; depth: 20 cm), and the samples were washed through a 2‐mm mesh sieve in the field. Bivalves were stored frozen in plastic bags and polychaetes were preserved in 70% ethanol until laboratory analysis. All organisms were identified, counted, measured (bivalves: length, height, width; polychaetes: length) and the ash‐free dry weight was determined as a measure of biomass (Beukema, 2017; Choi et al., 2017).

Sediments were sampled once at each station and the grain‐size fractions were analyzed.

Benthos and sediment data were used to interpret foraging patterns, but were not used for a detailed habitat modeling approach. For clarity, only the most abundant bivalves (length >= 20 mm) of foraging hotspots of herring gulls were shown.

### 2.4 Statistical analysis

All statistical analyses were performed using the open source software R 3.4.2 (R Development Core Team, 2017).

#### 2.4.1 Habitat choice based on GPS data

In this study, we only analyzed foraging trip data, that is all breeding positions on the nest and all trips targeting roosts were excluded from the analysis. The GPS fixes were classified as flying, roosting, breeding, or feeding behavior based on an analysis of the frequency distribution of the movement speeds of the birds (Shamoun‐Baranes et al., 2011; Yoda, Tomita, Mizutani, Narita, & Niizuma, 2012), as well as the geographical position (Schwemmer, Güpner, Adler, Klingbeil, & Garthe, 2016). All fixes with a speed >7 km/hr were defined as flying behavior. The start of a foraging trip was identified by an increase in speed and a subsequent change in geographical position. The first and last positions of each trip were either at the colony or at a resting site.

Trips with large gaps (≥30 min; *n* = 3) between consecutive positions were excluded from the analysis. Small gaps (<30 min) were interpolated linearly (R package data.table 1.10.4) to an interval of 2 min to allow comparisons of data collected using different logger types. Maximum trip distance (direct maximum distance), total trip distance (total distance flown per trip), and trip duration of foraging flights were calculated.

In addition to the trip characteristics, we also analyzed the foraging patterns in detail. To achieve this, GPS positions during flight were excluded from the dataset and the remaining GPS fixes were classified as land, mudflat, or sea, according to their geographical position. Visualization was performed using ArcGIS 10.1 (ESRI, 2011). Herring gulls from all three colonies foraged mainly on mudflats or on land, and only a few trips targeted the sea. Shamoun‐Baranes et al. (2011) demonstrated that birds could rest at sea for several hours, passively drifting with the tide. However, it is difficult to differentiate between resting and foraging at sea in the absence of accelerometer data, and because only a few positions targeted the sea, we excluded these positions and tested the differences in utilization of the two main habitats among the colonies.

Trip characteristics were analyzed using linear mixed‐effect models (LMM, R package lme4 1.1‐12) based on the restricted maximum likelihood estimation (Korner‐Nievergelt, 2015). To assess the effect of the geographical gradient on the foraging behavior of the herring gulls, we used each of the three trip parameters (total distance, maximum distance, and trip duration log‐transformed to achieve a normal distribution of the residuals) as dependent variables in separate LMMs, with colony as the only predictor. Bird ID was treated as a random factor in all LMMs, to account for pseudoreplication. For each model, we performed an analog model including only the random factor and tested for differences between both models using a likelihood ratio test (ANOVA; Faraway, 2006).

The influences of different predictors (colony location, water level, time of day, sex) on habitat choice (terrestrial vs. mud flat) were tested using binomial generalized linear mixed models with appropriate autoregression structures, using all available raw data.

Water level was measured every minute at stationary positions and obtained by Landesbetrieb für Küstenschutz, Nationalpark, und Meeresschutz Schleswig‐Holstein. Every GPS logger fix was assigned to the water level of the next measurement station (“Hilligenley”: 54°37′6.6″N, 8°32′50.424″E; “Langeness Kirchwarf”: 54°38′25.2096″N, 8°36′49.8528″E; “Pellworm Hafen”: 54°31′15.6144″N, 8°41′6.468″E; “Schluett”: 54°39′8.9748″N, 8°42′49.3272″E; “Wyk”: 54°41′36.4488″N, 8°34′34.5468″E).

According to the averaged sunrise and sunset times of the study days and region, hours from 22:00 to 04:49 Central European Summer Time (CEST) were classified as “night,” and hours from 05:00 to 21:59 CEST as “day.” To differentiate between the sexes, a breast feather was taken of the equipped birds for DNA analysis (females, *n* = 16; males, *n* = 17; unknown, *n* = 4). Laboratory work was conducted by Tauros Diagnostik GbR, Bielefeld, Germany.

The GPS‐tracking data were nested (especially trips within individuals) and highly temporally autocorrelated. To handle this extreme temporal autocorrelation, the raw data were thinned out using only every *N*‐th entry, starting at entry number *M* for every trip ID. Testing different values for *N* revealed that *N *=* *15 led to an autoregression‐order of approximately 2. However, the choice of *M* slightly influenced the final results due to stochasticity, and only part of the raw data were used for analysis. To address these problems, we fitted the final regression model separately to 14 different datasets (*M *=* *1, …, 14) and calculated the average values for the resulting regression coefficients, standard errors, degrees of freedom (*df*), and *t*‐values. The final *p* values were calculated based on these averages. To obtain and validate the optimal model, predictor selection was performed using the best‐subset method and based on the Akaike information criterion (AIC) value. A reasonable definition of the AIC based on mixed models is problematic (Korner‐Nievergelt, 2015; Zuur, Ieno, Walker, Saveliev, & Smith, 2009), and we therefore neglected the random effects solely for this predictor selection process, thus reducing the models to fixed effects. Model selection revealed that all the proposed predictors should be used as predictors within the final regression model. The final mixed‐effect model is thus given by the following equation:

(1) $$\begin{array}{cc} \mathrm{logit}\;(y_{\mathrm{ijk}})\; & =\;\beta_{0}\;+\;u_{i}\;+\;v_{j}\;+\;{\left(\mathrm{colony}\right)}_{k}\\ & \;+\;{\left(\mathrm{water}\_\mathrm{level}\right)}_{k}\;+\;{\left(\mathrm{day}\_\mathrm{night}\right)}_{k}\\ & \;+\;{\left(\mathrm{sex}\right)}_{k}+\epsilon_{\mathrm{ijk}}, \end{array}$$

with *ϵ*_*ijk*_ *~ N* (*0*,* σ* ^2^), *u*_*i*_ *~ N* (0, *σ*_*u*_^2^) and *v*_*j*_ *~ N* (0, *σ*_*v*_^2^). Here, *y*_*ijk*_ is the binary outcome, *i* and *j* refer to trip and bird ID, respectively, *k* refers to the observation number, and *u* and *v* represent the random intercepts related to trip and bird ID, respectively. Furthermore, *N* (*x*,*y*) indicates a normal distribution with mean *x* and variance *y*. Finally, model validation was performed by graphical analysis via various residual plots (Field, Miles, & Field, 2012; Zuur, 2012; Zuur, Ieno, & Elphick, 2010).

Odds ratios are given by the exponential of regression coefficients and indicate the change in odds of the binomial outcome variable resulting from a unit change in the predictor (Field et al., 2012). If the predictor is categorical, the corresponding odds ratio represents the change in odds of the considered factor level compared to the baseline level.

#### 2.4.2 Dietary analysis

Ratios of carbon and nitrogen isotopes were compared among colonies using a linear model. We applied Tukey's post hoc test to detect significant differences in stable isotope ratios among all colonies (Faraway, 2006; Korner‐Nievergelt, 2015).

## 3 RESULTS

### 3.1 Potential device effects and datasets

Thirty‐seven datasets from the 63 deployed loggers were available for analysis during incubation (Oland, *n* = 15; Langeness, *n* = 3; Amrum, *n* = 19, Table 1).

**Table 1:** Study location, study periods, logger type, and sample size of tagged herring gulls

Colony	Study period	No. of birds (available datasets)	No. of loggers from type “CatLog‐S”	No. of loggers from type “E‐obs”
Oland	7–25 May 2012	8	8	0
	7–20 May 2013	2	2	0
	14 May–1 June 2015	5	0	5
Langeness	11–28 May 2014	3	2	1
Amrum	14 May–4 June 2013	8	8	0
	7 May—1 June 2014	8	4	4
	16 May—3 June 2015	3	0	3

Most of the datasets covered >20 days, but three only covered 3–5 days because of battery problems. Data from 26 deployed loggers were not available for analysis: Five tagged individuals could not be recaptured, one had lost its device, five devices did not record any data, ten nests were predated by foxes, and five nests were destroyed by grazing cows, making it impossible to retrap the equipped birds. The predations by foxes and trampling of nests by cows led to a low breeding success of the whole colony, which included the tagged birds. This high rate of clutch loss meant that only three datasets were recorded for Langeness, demonstrating high individual variability in foraging patterns, and indicating that the results for this colony should be interpreted with caution.

Herring gulls equipped with CatLog‐S loggers had to be recaptured, and comparisons of the body mass at capture (mean ± *SD*: 982 ± 114) and recapture (mean ± *SD*: 987 ± 94) indicated an increase in average body mass. This indicated that the gulls were behaving and feeding normally. Individuals tagged with e‐obs loggers did not have to be recaptured. However, we observed five of these tagged birds and found no differences in behaviors (e.g., increasing preening activity or aggression against the attachments) compared with four control (untagged) birds (Schäfer‐Nolte, 2014).

### 3.2 Trip characteristics

**Figure 3:**
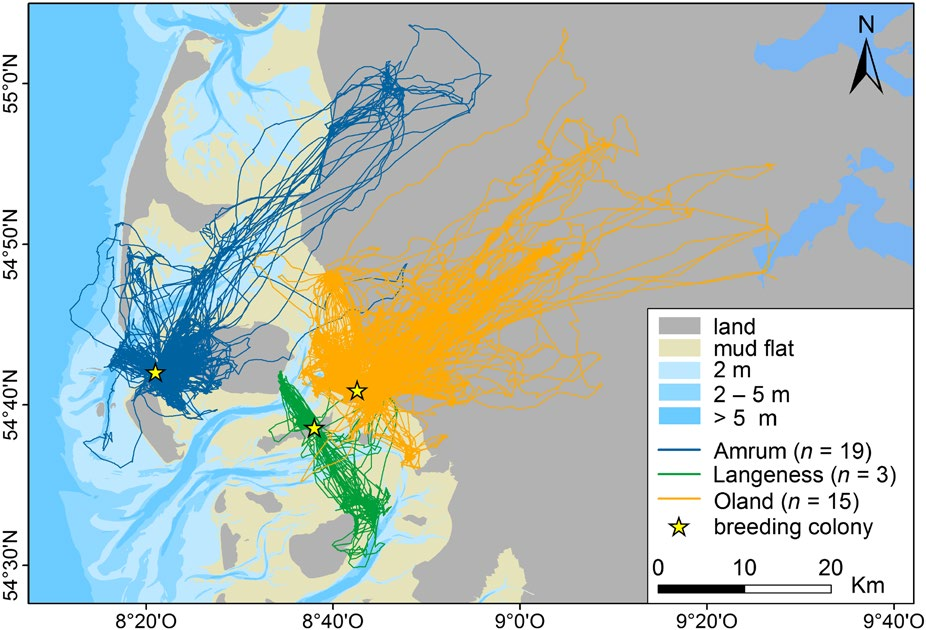
Foraging trips of tracked *Larus argentatus* (*n* = 37) breeding at three different colonies in the German North Sea (2012–2015)

Foraging trips (*n* = 1,115) differed substantially among the colonies (Oland: *n* = 407; Langeness: *n* = 80; Amrum: *n* = 628; Figure 3). Herring gulls from Amrum preferred to forage on tidal flats near the breeding colony, while relatively fewer trips were directed to the mainland or to terrestrial areas on the adjacent island of Föhr (67.5% exclusively targeted the tidal flats, 3.7% solely to terrestrial habitats, 28.8% mixed). Gulls from Langeness showed high individual variability and utilized both mudflats and terrestrial habitats on different Wadden Sea islands. For herring gulls breeding on Oland (the colony closest to the mainland), 37.1% of the foraging trips targeted the tidal flats, 20.6% targeted solely terrestrial habitats, and 42.3 % were mixed. Foraging trips often extended far inland (maximum distance from the nest = 54.6 km; Table 2) and also reached the coast of the Baltic Sea. The total and maximum distances of the foraging trips reflected the geographical gradient and differed significantly among the colonies (Table 2). Trips by Amrum individuals were shorter (mean = 12.3 km ± 8.4 km) compared with trips from Oland (mean = 26.7 km ± 26.1 km). The maximum distance travelled was higher for birds breeding at Oland than at Amrum, but the trip duration did not differ significantly among birds from the three colonies (Table 2). There were no sex differences in flight patterns.

**Table 2:** Total distance, maximum distance, and trip duration for herring gulls from different breeding colonies. Influence of colony on trip parameters was tested using likelihood ratio tests

	Total distance (km)	Maximum distance (km)	Trip duration (hr)
Oland	Mean = 26.7	Mean = 9.2	Mean = 5.22
	*SD* ± 26.1	*SD* ± 9.4	*SD* ± 5.26
	Min = 0.3	Min = 0.1	Min = 0.21
	Max = 148.8	Max = 54.6	Max = 37.30
Langeness	Mean = 21.6	Mean = 6.8	Mean = 5.09
	*SD* ± 18.2	*SD* ± 4.1	*SD* ± 3.38
	Min = 1.9	Min = 1.0	Min = 0.16
	Max = 83.8	Max = 16.6	Max = 190.26
Amrum	Mean = 12.3	Mean = 4.2	Mean = 3.32
	*SD* ± 18.4	*SD* ± 6.0	*SD* ± 4.17
	Min = 0.2	Min = 0.1	Min = 0.14
	Max = 190.3	Max = 51.6	Max = 45.27
Model	*χ*² = 7.6	*χ*² = 7.7	*χ*² = 3.9
	*df* = 2	*df* = 2	*df* = 2
	*p* = 0.022	*p *=* *0.022	*p *=* *0.143

### 3.3 Foraging patterns

The geographical position of the breeding colonies had a highly significant effect on the foraging habitat utilized; gulls breeding on the farthest offshore island, Amrum, foraged on the tidal flats five times more often than gulls breeding on Langeness (odds ratio [OR] = 0.2, *p* < 0.001) and Oland (OR = 0.17, *p* < 0.001), which were located closer to the mainland (Table 3).

**Table 3:** Averaged regression results for relative use of mud flats by herring gulls compared with Amrum, sex_female, and daytime. Final generalized linear mixed models applied to 14 datasets with different starting values *M* = 1,.., 14 for data thinning. For example, herring gulls breeding on Amrum foraged on the tidal flats five times more frequently than herring gulls from Langeness (=0.2 odds ratio)

	Beta	*SE*	*p*	Odds ratio
Langeness	−1.61	0.28	<0.001	0.20
Oland	−1.76	0.16	<0.001	0.17
Water level	−0.40	0.04	<0.001	0.67
Sex_male	−0.32	0.17	0.06	0.73
Night	0.59	0.08	<0.001	1.80

Presence at tidal flats was highly significantly associated with the tide, and herring gulls foraged in terrestrial areas more frequently in line with increasing water levels (OR = 0.67, *p* < 0.001). Herring gulls also foraged more intensively on tidal flats at night (OR = 1.8, *p* < 0.001) than during the day. There was no significant difference in the use of mud flats between females and males.

### 3.4 Dietary analysis

**Figure 4:**
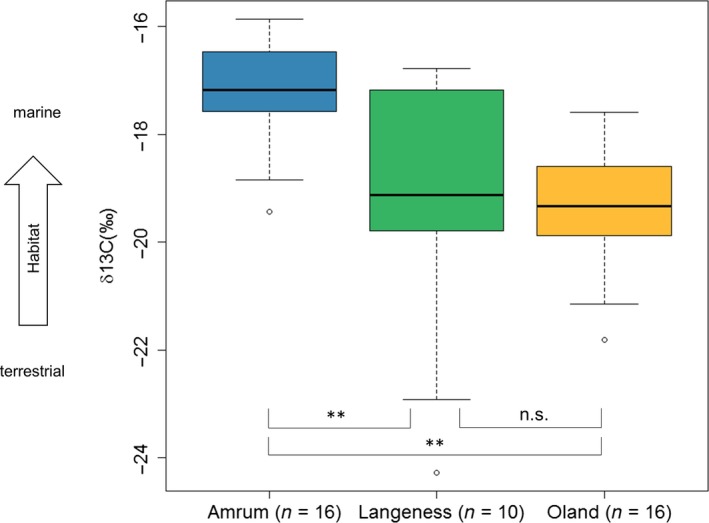
Carbon isotope ratios in red blood cells of herring gulls from breeding colonies on Amrum, Langeness, and Oland. Boxes represent 50% of the data between the first (25%) and the third (75%) quartile. Upper and lower whiskers represent maximum and minimum, respectively. Horizontal line marks the median. Level of significance: ***<0.001; **<0.01; *<0.05; n.s.: not significant

Stable isotope analysis of red blood cell samples showed a clear relationship between *δ* ^13^C and colony (*f* = 8.72, *df* = 2, *p* = 0.0007; Figure 4). Gulls breeding on Amrum had significantly higher *δ* ^13^C values than those from Oland (Tukey's test, *p* = 0.0016, 95% confidence interval [CI] = −3.4, −0.7) and Langeness (Tukey's test, *p* = 0.0058, 95% CI = −3.6, −0.5). There were no differences in *δ* ^13^C values between gulls from Oland and Langeness (Tukey's test, *p* > 0.95, 95% CI = −1.5, −0.5; Figure 4).

**Figure 5:**
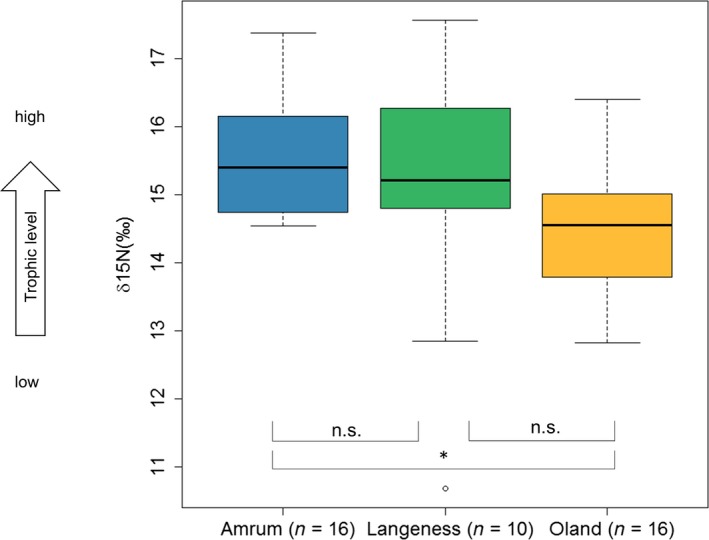
Nitrogen isotope ratios in red blood cells of herring gulls from breeding colonies on Amrum, Langeness, and Oland. For explanation of box plot, see Figure 4. Level of significance: ***<0.001; **<0.01; *<0.05; n.s.: not significant

*δ* ^15^N values also differed among all three colonies, but not significantly (*f* = 3.147, *df* = 2, *p* = 0.0541; Figure 5). Individuals from Amrum and Oland had different *δ* ^15^N values (Tukey's test, *p* = 0.04, 95% CI = −2.3, −0.03), but there was no difference in *δ* ^15^N values between gulls from Langeness and Amrum (Tukey's test, *p* = 0.62, 95% CI = −1.8, −0.8) or between Langeness and Oland (Tukey's test, *p* = 0.42, 95% CI = −1.98, −0.6; Figure 5). Compared with the other islands, birds from Langeness showed a higher variation in *δ* ^13^C and *δ* ^15^N values.

The mean numbers of food items per pellet were between one and two, and the maximum number of food items per pellet was five (Table 4). Herring gulls had a variable diet composition, ranging from earthworms and insects to bivalves, fish, and mammals. Herring gulls from Oland fed mostly terrestrially, while those from Amrum consumed prey originating from the intertidal flats (Table 4). The most important terrestrial prey items were earthworms (Table 4), while common cockles (*Cerastoderma edule*), razor clams (*Ensis leei*), and shore crabs (*Carcinus maenas*) were the most frequent prey items from mudflats, and swimming crabs (*Liocarcinus holsatus*) probably originating from the farthest offshore (habitat) zone were captured more frequently than fish. While diet composition of pellets from Amrum was similar over the years, herring gulls from Oland and Langeness showed a slightly more variable foraging behavior. The sample sizes were small for Oland and Langeness 2014 and should be interpreted with caution. Common cockles were mostly consumed and resulted in a higher proportion of intertidal prey items in 2012 of Oland gulls. Herring gulls from Langeness fed mostly in intertidal habitats, where razor clams, common cockles, and shore crabs were the most frequent prey items. In this colony, the proportion of swimming crabs was highest, and the proportion of prey items originating from the terrestrial habitat smallest in 2015 (Table 4).

**Table 4:** Semi‐quantitative frequency of each dietary component in pellets from herring gulls at different colonies during incubation. Sums of all prey items deviate from 100% due to rounding errors

	Oland (2012)	Oland (2013)	Langeness (2014)	Langeness (2015)	Amrum (2013)	Amrum (2014)	Amrum (2015)
*n* Pellets	22	35	26	114	56	123	85
Mean number of prey items	2	1	1	2	1	1	1
Max. number of prey items	5	5	3	5	4	5	4
Sea	4.5	12.9	7.7	17.4	17.5	11.1	9.6
Crustaceans (*Liocarcinus holsatus*)	0.9	11.7	6.5	12.8	15.2	10.9	8.6
Fish	3.6	1.1	1.2	4.6	2.3	0.2	1.1
Mudflat	55.5	33.4	50.0	59.4	74.5	78.0	79.2
Bivalves (*Mytilus edulis*)	3.6	4.0	0.0	3.9	5.2	2.4	0.0
Bivalves (*Ensis leei*)	12.3	11.4	11.5	17.5	18.2	11.8	3.9
Bivalves (*Limecola balthica*)	0.0	2.0	0.0	3.8	0.4	1.1	0.0
Bivalves (*Cerastoderma edule*)	22.7	0.3	22.7	12.8	19.3	32.6	47.3
Bivalves	0.0	1.4	0.0	0.3	0.4	0.0	0.0
Gastropods	0.0	0.0	0.0	1.5	0.0	0.0	0.0
Annelids (*Hediste diversicolor*)	0.0	0.0	0.0	0.0	0.5	0.1	0.7
Crustaceans (*Carcinus maenas*)	15.9	11.1	15.8	17.5	27.9	27.7	27.3
Crustaceans	0.9	3.1	0.0	2.0	2.7	2.3	0.0
Land	40.0	53.7	42.3	23.2	8.0	10.9	10.0
Earthworms	10.5	19.4	15.0	11.8	2.5	1.3	4.1
Insects	7.3	3.4	2.3	2.3	0.4	0.8	1.3
Birds	0.0	0.0	0.0	0.9	0.0	2.6	0.4
Eggs	4.5	14.3	15.4	2.6	0.0	0.8	2.4
Mammals	0.0	0.0	9.6	2.5	1.8	1.8	0.0
Plant material—crop/seed	17.7	16.6	0.0	2.2	3.4	2.0	1.9
Waste	0.0	0.0	0.0	0.9	0.0	1.6	0.0

### 3.5 Prey availability

Foraging hotspots of herring gulls breeding on Amrum had very high abundances (mean = 90.08) and biomass values (mean = 9.49 g) of common cockles, both in comparison with other bivalve species investigated and when compared to the other colonies. In the vicinity of Oland, only a small number of blue mussels and common cockles were available at the targeted hotspots. There, the dominant prey species was the razor clam with a mean abundance of 4.95 and a mean biomass of 13.03 g. In relation to their abundance, razor clams showed a high biomass (Table 5). Due to the small sample of logger data from herring gulls breeding on Langeness, only a few foraging hotspots could be identified for the tagged birds, with common cockle being the dominant bivalve species.

**Table 5:** Mean abundance (n/m^2^) and mean biomass (AFDW g/m^2^) of benthic prey as well as the mean mud content (63 μm) of sediment at foraging sites selected by herring gulls. For clarity, only the most abundant bivalves (length >= 20 mm) are shown

	Oland (*n* = 169 replicates)		Langeness (*n* = 9 replicates)		Amrum (*n* = 144 replicates)	
	n/m^2^ (*SD*±)	AFDW g/m^2^ (*SD*±)	n/m^2^ (*SD*±)	AFDW g/m^2^ (*SD*±)	n/m^2^ (*SD*±)	AFDW g/m^2^ (*SD*±)
Blue mussel (*Mytilus edulis*)	1.10 (±0.06)	0.10 (±0.01)			1.29 (±0.08)	0.42 (±0.03)
Razor clam (*Ensis leei*)	4.95 (±0.12)	13.03 (±0.39)			0.80 (±0.07)	0.03 (±0.00)
Baltic tellin (*Limecola balthica*)	2.20 (±0.08)	0.31 (±0.01)			5.32 (±0.17)	0.79 (±0.03)
Common cockle (*Cerastoderma edule*)	2.20 (±0.10)	0.80 (±0.04)	20.67 (±4.30)	1.58 (±0.34)	90.08 (±1.41)	9.49 (±0.15)
Mud content^a^ (%)	8.95 (±9.75)		26.38 (±31.27)		8.38 (±9.95)	

a Sediment was sampled per station: Oland *n* = 50; Langeness *n* = 3; Amrum *n* = 48.

Overall blue mussels were rarely found at the foraging hotspots.

The mud content of Langeness (26.38%) differed from Amrum (8.38%) and Oland (8.95%).

## 4 DISCUSSION

### 4.1 General foraging strategies

Both isotopic ratios and pellet data consistently supported the results from the loggers and indicated that herring gulls from Amrum, Oland, and Langeness had significantly different foraging patterns during incubation season.

The carbon signals in particular were significantly correlated with the distance of the breeding location from the mainland, such that gulls breeding farther offshore had a higher carbon and nitrogen signal (indicating a more intensive use of marine/intertidal prey) than birds breeding closer to the coast.

Goss‐Custard (1977) and van Gils, Spaans, Dekinga, and Piersma (2006) showed that birds adapted their foraging strategies according to the energy‐intake rate at the foraging site and the distance between the foraging and roosting areas. Gulls from Amrum, the colony farthest from the mainland, made the shortest foraging trips and spent the highest proportion of time feeding in intertidal areas, relative to gulls from the other colonies. Observations and benthos sampling revealed that the birds from Amrum visited a large cockle bed close to the breeding colony as a feeding habitat. This area was exposed for a long time during low tide and had a high biomass of potential prey. Pellets from herring gulls from Amrum included the highest proportion of intertidal prey, relative to gulls from other colonies. Similar to the 1990s (Garthe, Freyer, Hüppop, & Wölke, 1999; Kubetzki & Garthe, 2003), common cockles and shore crabs were the dominant prey items, although razor clams were more frequent in 2013–2015. Terrestrial prey items were the least common in the pellets.

The location of Amrum close to the open sea suggested that herring gulls might follow fishing vessels to obtain easily accessible prey, but data from loggers and pellets did not support this idea, given that only a few trips targeted the sea.

Pelagic prey is energetically rich but unpredictable, due to its patchy distribution (Garthe & Hüppop, 1994; van Donk et al., 2017; Weimerskirch, 2007), while terrestrial and intertidal habitats are characterized by more stable feeding conditions (reliability, low handling and flight costs). The logger data implied that predictable and productive intertidal habitats, such as the cockle bed, were well known to the breeding herring gulls and were thus used intensively for foraging.

In contrast, herring gulls breeding on Oland, closest to the mainland, carried out the longest foraging trips and spent more time in terrestrial habitats compared with birds from the other colonies. Only a few trips targeted defined intertidal areas near the breeding colony.

Previous studies demonstrated that flights over land consumed less energy than expected, and flight costs were reduced using fine‐scale structures (e.g., dykes) to increase orographic lift (Shamoun‐Baranes, Bouten, van Loon, Meijer, & Camphuysen, 2016; Shepard, Williamson, & Windsor, 2016). Furthermore, birds prefer foraging habitats with predictable prey distributions during the breeding season (Camphuysen, 2013; Weimerskirch, 2007), as expected in terrestrial habitats (Gorke & Brandl, 1986; Palm, van Schaik, & Schröder, 2013; Sibly & McCleery, 1983). The energetic contents of terrestrial and intertidal prey items have been shown to be comparable (Cummins & Wuycheck, 1971), which suggest that terrestrial foraging should be as beneficial as marine foraging, particularly for gulls breeding in a colony close to the mainland. However, herring gulls foraging at higher trophic levels have demonstrated better body conditions and a higher breeding success (O'Hanlon et al., 2017), as well as a higher lifetime reproductive success (van Donk et al., 2017). Although no breeding success data were measured in our own study, the results of these previous studies suggest that terrestrial food resources may not be particularly disadvantageous as often assumed. However, this depends much on the type of terrestrial food and apparently intertidal habitats remain more important for foraging and reproductive success.

### 4.2 Influence of tide, time of day, and density dependence

Intertidal systems are characterized by their elevation and their inundation time (Evans, 2002), which influence the biomass and species richness of suitable prey (Beukema, 1976; Waser et al., 2016; Yates et al., 1993). Prey depletion close to the breeding colony may be counteracted by a habitat switch (Schwemmer & Garthe, 2008) or a change in the diet (Goss‐Custard et al., 2006; O'Connor & Brown, 1977; Zwarts & Wanink, 1993). A previous study on the food choice of oystercatchers noted a shortage of suitable prey in the vicinity of Oland (Schwemmer et al., 2016). In our study, benthos samples at foraging hotspots confirmed this shortage, especially of common cockles. Herring gulls may thus have switched their foraging strategy to a mainly terrestrial one, in addition to learning to utilize newly introduced species in the intertidal zone as a novel prey resource. Inspection of important spots used by the tagged birds revealed that herring gulls from Oland visited razor clam beds in the intertidal zone, although these were only available to herring gulls for a short time during low tide. Razor clams were accidently introduced to the Wadden Sea with ballast water in 1978 (Dannheim & Rumohr, 2012; Freudendahl, Nielsen, Jensen, & Jensen, 2010; von Cosel, Dörjes, & Mühlenhardt‐Siegel, 1982), and the species has since extended its range substantially over the last three decades (Dekker & Beukema, 2012). Common eiders, common scoters, and various shorebird and gull species regularly use this new source of food (Swennen, Leopold, & Stock, 1985; Tulp et al., 2010). Compared with other bivalve species, razor clams represent an energy‐rich prey organism with a good shell–flesh‐ratio (own unpublished data).

We regularly recorded herring gulls feeding at razor clam beds, with some individuals transporting razor clams to their breeding colony, but most birds opening the shells and only consuming the flesh, suggesting that razor clams are likely to be underrepresented in pellets.

However, razor clams and other bivalve beds are only available for foraging for a short time during the lowest part of the tide, and herring gulls were forced to use the mainland as a feeding habitat at other times. Logger data for herring gulls breeding on, for example, Oland showed a high proportion of mixed foraging trips, suggesting that individuals from this colony might be forced to forage on the mainland after the razor clam beds become inundated by the tide. Herring gulls are flexible and opportunistic predators that can adapt their diet to the habitat (Pierotti & Annett, 1991; Schwemmer & Garthe, 2008; Sibly & McCleery, 1983) and might act as an indicator for changes in prey availability (Courtens, Verstraete, Vanermen, Van de Walle, & Stienen, 2017) close to their breeding colony during incubation.

In addition to the tidal cycle, the foraging strategy is also strongly influenced by the time of day. The relative use of mud flats highly significantly increased at night compared to the daylight period. Herring gulls are both visual and tactile foragers, and they may optimize their food intake (Yoda et al., 2012) by avoiding terrestrial habitats at night to minimize the risk of predation by foxes and other mammalian predators, while it might be equally feasible to search for food in the dark on mud flats as compared to daylight.

### 4.3 Competition and influence of sex

Pellet analysis revealed a wide spectrum of prey items. Although the logger data showed that different habitat types were often visited in the same foraging trip, the prey diversity within a single pellet was relatively small. Overall, the dietary niche of herring gulls overlaps with that of other gull species, such as common and black‐headed gulls, both of which are also concentrated in coastal areas and forage in intertidal and terrestrial habitats (Kubetzki & Garthe, 2003). Earthworms have also been shown to be an important food source for lesser black‐backed gulls breeding inland and at the coast, which also forage on the mainland (Coulson & Coulson, 2008; Garthe et al., 2016).

Inter‐ and/or intraspecific competition (Corman et al., 2016; Hamilton, Gilbert, Heppner, & Planck, 1967) may thus be an important factor driving decisions on where to forage. Corman et al. (2016) demonstrated low inter‐ and intracolonial overlaps in foraging behavior in lesser black‐backed gulls. In our study, gulls from Langeness were the most flexible in terms of habitat choice although the sample size of tagged birds from this colony was very low. Both terrestrial and intertidal areas were easily accessible to these birds because of the central location of the breeding colony. However, this colony location could also imply greater intra‐ and interspecific competition with other colonies. Herring gulls from Langeness might thus choose flexible foraging sites to avoid intraspecific competition from neighboring colonies (e.g., Oland, Amrum, Föhr), as well as intraspecific competition from within the same colony. Although only three datasets were available for Langeness, 80 foraging trips from these three individuals were included in the analysis. Nevertheless, the high individuality of herring gulls breeding on Langeness might lead to different results with a higher sample size. Although the logger dataset for Langeness was small, stable isotope and pellet analyses supported a flexible foraging strategy for birds from this colony. The same could be supposed for herring gulls breeding closer to the coast. Foraging trips heading to the west might not be an option for gulls from Oland, because of intraspecific competition from neighboring colonies (Corman et al., 2016).

We found no sex‐related differences in foraging trip characteristics during incubation. However, lesser black‐backed gulls have demonstrated sexual segregation in foraging patterns throughout incubation and chick rearing (Camphuysen, Shamoun‐Baranes, van Loon, & Bouten, 2015), with males traveling longer distances from the colony and feeding mostly on fisheries discards. Camphuysen et al. (2015) suspected that the marginally larger males foraged more successfully behind fishing vessels compared with the smaller females. In contrast, herring gulls in the current study predominantly targeted the vast intertidal and terrestrial habitats for foraging, where sexual dimorphism is less likely to confer any advantage because prey is usually distributed much more evenly over a wide area.

## 5 CONCLUSIONS

Herring gulls in our study showed a wide range of foraging behaviors, from intertidal to combined intertidal–terrestrial strategies. This switch was driven by the distance between the colony and the mainland, the level of the tide, the time of day, and the food availability near the breeding colony.

Logger data, as well as stable isotope and pellet analyses, confirmed a geographical gradient in terms of foraging behaviors during incubation; gulls breeding close to the mainland mainly used terrestrial habitats as foraging grounds, while gulls breeding farther offshore preferred intertidal areas close to the breeding colony. Behavioral observations, and analyses of pellet and logger data demonstrated that herring gulls utilized the nonindigenous species *Ensis leei* as new prey source. The logger data also implied that predictable and productive intertidal habitats, such as cockle beds and razor clam fields, were well known to the breeding herring gulls and were utilized for foraging, although the birds were forced to feed in terrestrial areas at high tide. Furthermore, herring gulls preferred to forage on mud flats at night and in terrestrial habitats during the day (and interestingly very similar to lesser black‐backed gulls which also use terrestrial habitats during the daylight but often switch to pelagic habitats at night (Garthe et al., 2016)). Prey depletion during incubation near the breeding colony might have increased the degree of foraging in terrestrial habitats. Given that herring gulls prefer to forage on bivalve beds, these gulls can be used to assess the status of the intertidal system close to their breeding colonies and may thus act as indicators of changes in the food web during incubation.

## CONFLICT OF INTEREST

The authors state that they have no conflict of interest.

## DATA ACCESSIBILITY

Tracking data are archived at Movebank (https://www.movebank.org). Bird‐ringing data are archived at the Beringungszentrale Vogelwarte Helgoland (Institute for Avian Research, Wilhelmshaven).

## AUTHOR CONTRIBUTIONS

LE, PS, and SG designed the study. LE and PS conducted fieldwork. LE and CCV performed laboratory work. LE, PS, and AMC analyzed the data. LE wrote the manuscript, to which all other authors contributed revisions.
